# Conservative esthetic management of post orthodontic treatment discolored tooth with calcified canal: a case report

**DOI:** 10.11604/pamj.2020.37.254.21982

**Published:** 2020-11-19

**Authors:** Muhammad Qasim Javed, Sumyya Saleh, Hamza Ulfat

**Affiliations:** 1 Department of Conservative Dental Sciences and Endodontics, College of Dentistry, Qassim University, Buraydah, Saudi Arabia,; 2 Department of Operative Dentistry, Riphah International University, Islamabad, Pakistan

**Keywords:** Calcific metamorphosis, root canal, pulp canal obliteration, case report

## Abstract

A case of pulp canal obliteration (PCO) two years after the completion of orthodontic treatment is presented. Post orthodontic treatment PCO is a rare finding. A 23 years old female presented with the discoloration of clinical crown of maxillary right central incisor. Radiographic examination revealed the calcified canal and diffused periapical radiolucency. Vitality tests were negative. A decision was made to do root canal treatment followed by the walking bleaching. After the successful completion of the root canal treatment the internal bleaching was performed. The discolored tooth showed significant improvement in color. Internal bleaching is a viable conservative treatment for improving esthetics in single non vital discolored tooth.

## Introduction

Calcific metamorphosis or pulp canal obliteration (PCO) is the pulpal response to trauma, characterized by rapid deposition of mineralized tissue in the root canal space. An array of factors such as dental trauma, carious lesions, abfraction, abrasion, pulp capping, occlusal imbalance, orthodontic treatment, harmful oral habits and natural aging can trigger PCO. The PCO is becoming increasingly common [1]. The presence of pulp necrosis after the PCO is reported in up to 30% of the cases [2]. Approximately 4-24% of the traumatized teeth develop varying degree of pulpal obliteration that is characterized by the apparent loss of pulp space radiographically and yellowish discoloration of crown [3]. Moreover, PCO increases the complexity level of the endodontic treatment and lateral/apical ramification of the main root canal create potential pathways through which bacteria can spread in the periapical area and remain unaffected by treatment procedures [4]. Post orthodontic treatment, complete PCO is a rare finding. Here we report the case of PCO following the completion of orthodontic treatment, its endodontic and esthetic management.

## Patient and observation

**Figure 1:**
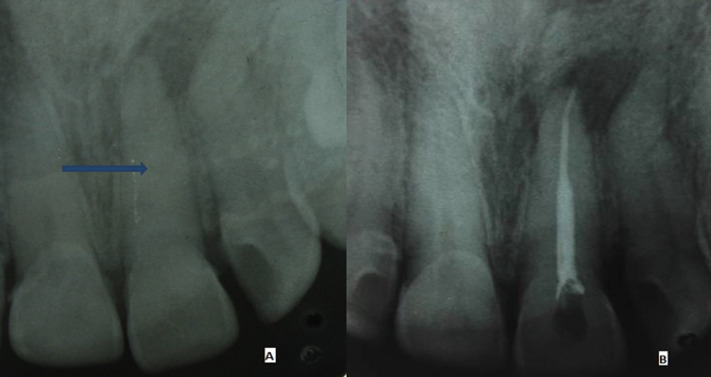
periapical radiographs of maxillary right central incisor: A) preoperative: calcified canal (blue arrow); B) obturation

A 23 year old female patient presented with the chief complaint of discolored maxillary right central incisor. Patient gave the history of malaligned maxillary right central incisor and subsequent orthodontic treatment that was finished two years ago. The response to vitality tests was negative. On percussion and palpation test, patient reported mild pain. Digital periapical radiograph revealed right maxillary central incisor with obliterated canal and diffuse periapical radiolucency (Figure 1A). The tooth had yellowish grey crown. Periodontal pocket depth was normal. A diagnosis of pulp necrosis and calcific metamorphosis was reached. After giving anesthesia and isolation, an endodontic access cavity was prepared at upper right central incisor with number 2 round diamond bur (Mani, Inc. Japan). The slit of the pulp chamber became visible and calcification was removed by using ultrasonic tip. Canal orifice was located with the help of endodontic explorer (DG-17). Patency of the canal was achieved with 08 K-hand file (Mani, Inc. Japan). The working length was measured with the radiographic method. After preparation of glide path canal was cleaned and shaped to size 30/.04 by using hyflex© CM file system (coltene). Irrigation was done by utilizing 3% sodium hypochlorite (septodont) and EDTA 15% (septodont), alternatively, with rinse of saline in between. The 27 gauge closed end side vent needle was used for irrigation. The canal was dried with sterile paper points. Obturation was done with Gutta Percha (GP) points and calcium hydroxide based sealer (sealapex, sybron endo) by using cold lateral condensation technique (Figure 1B).

**Figure 2:**
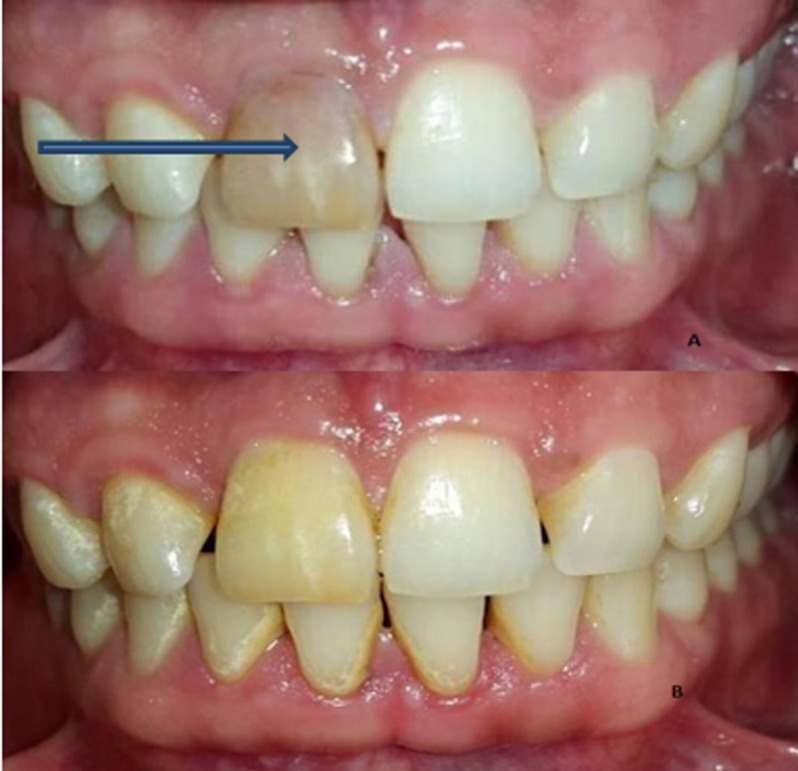
A) preoperative picture with discolored tooth (blue arrow); B) postoperative picture

Subsequently, GP was removed 2-3mm apical to canal orifice and restorative glass ionomer cement (GIC) was applied on the obturating material to minimize leakage of bleaching agent. After placing rubber dam 35% hydrogen peroxide (opalescence® endo® ultradent gel) was injected into the chamber for the management of yellowish grey crown (Figure 2A), followed by the placement of cotton pellet and sealing of access cavity with restorative GIC (vitremer, 3M). The process was repeated three times after the interval of five days. Desired results were achieved after the third visit and patient was satisfied (Figure 2B). Subsequently, the tooth was permanently restored with resin composite after 7 to 10 days.

## Discussion

The psychosocial well-being of a person can be influenced by smile attractiveness. Tooth color has particular cosmetic importance and is readily perceived by people. In many cases just one discolored tooth can compromise the entire smile harmony [5]. Furthermore, accurate diagnosis of the etiology of discoloration is important prerequisite for conducting an adequate treatment [6]. This case report describes the management of anterior calcified and discolored tooth subsequent to orthodontic therapy. As compared to other complications of orthodontic treatment like root resorption, gingival recession and white spot lesions, tooth discoloration is a rare phenomenon; however, it may come to patients as a disturbance due to necrosis of pulp that may influence the doctor patient relationship [7]. The yellowish grey discoloration is a common finding in teeth with pulp canal obliteration but does not imply the presence of pulpal or periapical disease. Root canal treatment is indicated when there are clinical signs/symptoms and radiographic findings suggestive of periapical disease. However, root canal treatment of a calcified canal can challenge the skills of practitioners. Procedural errors can arise from overzealous inappropriate attempts to locate the canal. Large pulp stone can be dissected out of an access cavity using burs, but preferably by using special ultrasonic tips. Accordingly, in the present case a specific ultrasonic tip, start X™ #3 (dentsply maillefer) had been used which was meant for scouting of the calcified canal.

After endodontic procedure, intracoronal bleaching of discolored non-vital tooth offers advantages over more conventional treatments, veneers and crowns. Perhaps the most significant advantage is the conservation of the natural tooth structure. However, bleaching can have adverse effects. Possible localized adverse effects are on dental hard tissue/mucosa and risk of external cervical resorption. However, studies on vital teeth have shown that adverse effects appear not to be permanent [8]. Recently, carbamide peroxide has been recommended for use in intracanal bleaching. The study reported that 35% carbamide peroxide showed the lowest levels of extraradicular diffusion [9]. However, literature has supported the use of 35% hydrogen peroxide as a bleaching agent after placing cervical barrier and without heat activation for faster and long-term effects. Rotstein *et al*. [10] demonstrated that a 2mm layer of glass ionomer cement (GIC) was effective in preventing the diffusion of 30% hydrogen peroxide solution. Thus, the GIC should be used as a base during walking bleaching, later it can be left in place after bleaching and serve as a base for final restoration [11]. Moreover, studies have reported the enhanced efficacy of hydrogen peroxide for bleaching when compared with carbamide peroxide and sodium perborate. Hydrogen peroxide breaks down faster than carbamide peroxide, so it releases most of its whitening power within 30-60 minutes. Regarding the effects of the use of light source, there is no evidence that its use to activate the hydrogen peroxide enhances the whitening of tooth. However, 30% hydrogen peroxide that is caustic, in combination with heat source is likely to initiate the cervical resorption [11]. Therefore, heat use was avoided in the current case.

## Conclusion

The root canal treatment along with the walking bleach technique using 35% hydrogen peroxide can provide good results in discolored tooth with pulp canal obliteration. However, 2mm protective base as a barrier must be placed to avoid the initiation of external cervical root resorption.

## References

[1] Soares de Toubes KM, Drummond de Oliveira PA, Machado SN, Pelosi V, Nunes E, Silveira FF (2017). Clinical approach to pulp canal obliteration: A case series. Iran Endodontic Journal.

[2] Moura LB, Velasques BD, Silveira LFM, Martos J, Xavier CB (2017). Therapeutic Approach to Pulp Canal Calcification as Sequelae of Dental Avulsion. Eur Endod J.

[3] Mccabe PS, Dummer PMH (2012). Pulp canal obliteration: an endodontic diagnosis and treatment challenge. Int Endod J.

[4] Chaniotis A, Filippatos CG (2017). The use of a novel approach for the instrumentation of a cone beam computed tomography-discernable lateral canal in an unusual maxillary incisor: case report. J Endod.

[5] Dunn WJ, Murchison DF, Broome JC (1996). Esthetics: Patients Perceptions of Dental Attractiveness. J Prosthodont.

[6] Watts A, Addy M (2001). Tooth discoloration and staining: a review of the literature. Br Dent J.

[7] Baik U-B, Kim H, Chae HS, Myung J, Chun Y (2017). Teeth discoloration during orthodontic treatment. The Korean Journal of Orthodontics.

[8] Plotino G, Buono L, Grande NM, Pameijer CH, Somma F (2008). Nonvital Tooth Bleaching: A Review of the Literature and Clinical Procedures. J Endod.

[9] Lee GP, Lee MY, Lum SOY, Poh RSC, Lim KC (2004). Extraradicular diffusion of hydrogen peroxide and pH changes associated with intracoronal bleaching of discoloured teeth using different bleaching agents. Int Endod J.

[10] Rotstein I, Zyskind D, Lewinstein I, Bamberger N (1992). Effect of different protective base materials on hydrogen peroxide leakage during intracoronal bleaching in vitro. J Endod.

[11] Féliz-Matos L, Hernández LM, Abreu N (2015). Dental Bleaching Techniques; Hydrogen-carbamide Peroxides and Light Sources for Activation, an Update. Mini Review Article. Open Dent J.

